# Inaugural Message from the Founding Editor-in-Chief

**DOI:** 10.15761/JTS.1000101

**Published:** 2015-05-28

**Authors:** Kenneth Maiese

**Affiliations:** Cellular and Molecular Signaling, USA

Given the remarkable advances made over the last several decades in science and medicine, there could be no better and opportune time to launch the *Journal of Translational Science* (*JTS*). We have been fortunate to witness the integration of multiple areas of discovery in science and medicine to push the envelope for the diagnosis and treatment of disorders that can affect any region of the body. In developed countries such as the United States, life expectancy continues to increase and is accompanied by a one percent decrease in the age-adjusted death rate from the years 2000 through 2011. Yet, with the increasing growth and lifespan of the world's population, it is expected that the incidence of a number of disorders, such as metabolic disease, and neurodegenerative disorders, also will continue to grow. For example, the World Health Organization reports that diabetes mellitus will be ranked as the seventh leading cause of death by the year 2030.

As a result, multiple novel therapeutic strategies are currently being advanced to treat disorders that currently have limited remedies. In particular, stem cell applications have quickly advanced and are being considered for numerous disorders that include cardiovascular disease, diabetes mellitus, cancer, inflammatory bowel disease, peripheral ischemia, graft versus host disease, and nervous system disorders. More than 40 years prior, the use of bone marrow transplantation set the stage for the treatment of hematological disorders and cancer with the use of stem cells. Presently, more than 50,000 bone marrow transplants are performed every year using autologous and allogeneic hematopoietic stem cells. In addition, unique cellular channels, such as the vanilloid (capsaicin) receptor TRPV1 (transient receptor potential, vanilloid subfamily member 1) and a member of the TRP superfamily of cation channels, are being pursued for a diverse spectrum of disorders that range from pain management to neuronal protection of the brain following stroke. Increasing consideration also is being directed to the field of microRNAs (miRNAs). MiRNAs consist of 19-25 nucleotides, are small non-coding ribonucleic acids (RNAs), and can control gene expression by silencing targeted messenger RNAs (mRNAs) translated by specific genes. With the explosion of new studies, miRNAs are reported to play critical roles in stem cell renewal, tissue repair, and potential disease progression in areas such as those of the cardiac, gastrointestinal, and nervous systems.

Although these therapeutic strategies appear initially to be diverse, upon closer inspection each depends upon a “common signaling platform” that is intimately connected through cellular pathways that can significantly affect clinical outcome in patients. It is clear that cellular and genetic targets can easily impact multiple disease entities. The translation of these pathways into relevant clinical treatments can yield fruit for many disorders. *JTS* was conceived with this knowledge in mind. *JTS* is a novel open access journal that will focus upon the translation of cellular, molecular, and genetic pathways into clinical strategies for multiple medical disciplines that can impact a broad spectrum of disorders. These disorders can involve all aspects of translational research and medicine to include stem cells, degenerative diseases, aging, immune function, tumorigenesis, epigenetics, musculoskeletal function, cognition, behavior, metabolic function, and targets of the neuronal, cardiac, pulmonary, gastrointestinal, and vascular systems. *JTS* will provide a platform in today's scientific and medical literature to serve as an international forum for the healthcare and scientific communities worldwide to translate basic and clinical research studies into clinical therapies as well as report upon prognostics, novel therapeutic strategies, and biomarker development. *JTS* will offer multiple venues to present authors' thoughts and investigations as original research papers, review papers, clinical studies, editorials, expert opinion and perspective papers, commentaries, book reviews, and letters to the editor.

Ultimately, *JTS* will promote a rigorous, fair, and constructive evaluation of all submitted articles. Papers will be processed in an extremely timely fashion so that all authors can follow the progress of the papers during the evaluation, copyediting, and publication phases. We are extremely excited with the launch of *JTS* and believe that the journal will fill a great void in the reporting of today's scientific and medical discoveries. *JTS* will serve as an international forum for the greatest degree of scholarship in the research and clinical communities worldwide to translate pioneering “bench to bedside” breakthroughs into successful clinical strategies. On behalf of the Editorial Team and the Editorial Board, welcome to *JTS*!

